# Effectiveness and safety of oral anticoagulants in older adults with non-valvular atrial fibrillation and heart failure

**DOI:** 10.1371/journal.pone.0213614

**Published:** 2019-03-25

**Authors:** Alpesh Amin, Alessandra B. Garcia Reeves, Xiaoyan Li, Amol Dhamane, Xuemei Luo, Manuela Di Fusco, Anagha Nadkarni, Keith Friend, Lisa Rosenblatt, Jack Mardekian, Xianying Pan, Huseyin Yuce, Allison Keshishian

**Affiliations:** 1 University of California, Irvine, California, United States of America; 2 University of North Carolina, Chapel Hill, North Carolina, United States of America; 3 Bristol-Myers Squibb Company, Lawrenceville, New Jersey, United States of America; 4 Pfizer, Inc., Groton, Connecticut, United States of America; 5 Pfizer Inc., New York, New York, United States of America; 6 Bristol-Myers Squibb Company, Wallingford, Connecticut, United States of America; 7 New York City College of Technology, City University of New York, New York, New York, United States of America; 8 SIMR, Inc, Ann Arbor, Michigan, United States of America; Universita degli Studi di Napoli Federico II, ITALY

## Abstract

Direct oral anticoagulants (DOACs) are at least as efficacious and safe as warfarin among non-valvular atrial fibrillation (NVAF) patients; limited evidence is available regarding NVAF patients with heart failure (HF). US Medicare enrollees with NVAF and HF initiating DOACs (apixaban, rivaroxaban, dabigatran) or warfarin were selected. Propensity score matching and Cox models were used to estimate the risk of stroke/systemic embolism (SE), major bleeding (MB), and major adverse cardiac events (MACE) comparing DOACs versus warfarin and DOACs versus DOACs. We identified 10,570 apixaban-warfarin, 4,297 dabigatran-warfarin, 15,712 rivaroxaban-warfarin, 4,263 apixaban-dabigatran, 10,477 apixaban-rivaroxaban, and 4,297 dabigatran-rivaroxaban matched pairs. Compared to warfarin, apixaban had lower rates of stroke/SE (hazard ratio = 0.64, 95% confidence interval = 0.48–0.85), MB (hazard ratio = 0.66, 0.58–0.76), and MACE (hazard ratio = 0.73,0.67–0.79); dabigatran and rivaroxaban had lower rates of MACE (hazard ratio = 0.87,0.77–0.99; hazard ratio = 0.84, 0.79–0.89, respectively). Rivaroxaban had a lower stroke/SE rate (hazard ratio = 0.65, 0.52–0.81) and higher MB rate (hazard ratio = 1.18, 1.08–1.30) versus warfarin. Compared to dabigatran and rivaroxaban, apixaban had lower MB (hazard ratio = 0.71, 0.57–0.89; hazard ratio = 0.55, 0.49–0.63) and MACE rates (hazard ratio = 0.80, 0.69–0.93; hazard ratio = 0.86, 0.79–0.94), respectively. All DOACs had lower MACE rates versus warfarin; differences were observed in stroke/SE and MB. Our findings provide insights about OAC therapy among NVAF patients with HF.

## Introduction

Atrial fibrillation (AF), the most common cardiac dysrhythmia in the United States, increases the risk of stroke 5-fold and is associated with 15–20% of all strokes [1], ultimately leading to a higher risk of functional or neurological deficits and a higher mortality rate [2,3]. Non-valvular AF (NVAF) and heart failure (HF) are some of the most common cardiac conditions, and they often coexist [4]. The prevalence of HF among NVAF patients ranges from 27–64% in randomized control trials [5–8] and 21–48% in real-world studies [9–11]. Both conditions affect the elderly and share many of the same risk factors, which synergistically increase the risk of stroke/systemic embolism (SE) [12–14].

Anticoagulation is recommended for patients with AF and concomitant HF for the prevention of stroke/SE [15]. Randomized control trials have shown that direct oral anticoagulants (DOACs) are at least as efficacious and safe as warfarin [16]. HF is associated with reduced time in therapeutic range [17], which results in increased risk of stoke/SE. However, post-hoc randomized control trial analyses found no significant interaction between the treatment effect of DOACs versus warfarin and HF status among AF patients [5–8]. Meta-analyses of randomized control trials have shown that DOAC use reduced the risk of stroke/SE, major bleeding (MB), and intracranial hemorrhage compared to warfarin among patients with NVAF and HF; the results were consistent among patients with and without HF [18,19].

Few observational studies have evaluated outcomes of DOACs versus warfarin use among NVAF patients with HF in a real-world setting [9,20,21]. Prior studies examined safety outcomes, but limited comparison information is available regarding the effectiveness of all OACs. A recent observational study demonstrated that patients prescribed DOACs had lower all-cause mortality compared to those prescribed vitamin K antagonists; however, the study was limited by sample size [21]. Further, no randomized control trials or real-world studies have evaluated the efficacy and safety of DOACs versus DOACs among NVAF patients with HF.

Therefore, we conducted a real-world observational study to compare the risk of stroke/SE, MB, and MACE among NVAF patients with HF prescribed each DOAC (apixaban, dabigatran, or rivaroxaban) versus warfarin and between DOACs in a Medicare-enrolled population.

## Materials and methods

This was a retrospective observational analysis using US fee-for-service Medicare data from the Center for Medicare and Medicaid Services, from January 01, 2012 through September 30, 2015. Medicare provides health insurance coverage for >38 million people aged ≥65 years as well as for those with end-stage kidney disease or a disability. Medicare data captures comprehensive demographic and clinical information using enrollment records as well as International Classification of Diseases, 9^th^ Revision, Clinical Modification, Healthcare Common Procedure Coding System codes, and National Drug Codes. This observational study was conducted under the provisions of Privacy Rule 45 CFR 164.514(e). The study was exempt from institutional review board review and approval because there was no collection or use of personally identifiable information in the conduct of this study [22].

Elderly patients (≥65 years) with ≥1 pharmacy claim for apixaban, dabigatran, edoxaban, rivaroxaban, or warfarin during the identification period (01JAN2013-31SEP2015) were selected. For patients with a DOAC prescription, the first DOAC pharmacy claim date during the identification period was designated as the index date. The first warfarin prescription date was designated as the index date for patients prescribed only warfarin and without any DOAC claim. Patients were required to have continuous medical and pharmacy health plan enrollment for ≥12 months prior to and on the index date as well as an AF and HF diagnosis during the 12 months prior to or on the index date (S1 Table) [23].

Patients were excluded from the study if they had a diagnosis of valvular heart disease, venous thromboembolism, transient AF, cardiac surgery, or a pharmacy claim for an anticoagulant during the 12 months prior to or on the index date; pregnancy during the study period; or hip/knee replacement within 6 weeks prior to the index date. Additional exclusion criteria can be found in S1 Fig.

Patient data were assessed from the day after the index date until the earliest of the following: 30 days after the discontinuation date, treatment switch, death, end of continuous medical and pharmacy enrollment, or end of the study period (September 30, 2015). A 30-day gap in prescription was used to define treatment discontinuation. A switch was defined as a prescription for an OAC other than the index OAC during the follow-up period, and the switch date was the new OAC prescription date within ±30 days of the last days’ supply of the index OAC.

Baseline variables included demographic variables for age, sex, race, socioeconomic status (Medicaid-dual eligibility, Part D low-income subsidy, and socioeconomic status score—a proxy score based on income, education, and occupation associated with each ZIP code) [24,25], and clinical characteristics such as clinical risk scores, prior stroke/SE, prior bleeding, comorbidities, baseline medication use, baseline hospitalization, and index dose.

The primary outcomes were stroke/SE, MB, and MACE. Stroke/SE included ischemic stroke, hemorrhagic stroke, and SE; MB included gastrointestinal bleeding, intracranial hemorrhage, and other MB sites; and MACE included stroke, myocardial infarction, and all-cause mortality. Clinical outcomes were determined using the primary diagnosis on discharge records from hospitalizations (S1 Table) and based on validated administrative-claim based algorithms [26,27]. Death was obtained by validated Social Security records that include the date of death. The study was registered at clinicaltrials.gov (https://clinicaltrials.gov/ct2/show/NCT03508271).

Descriptive analysis of clinical and demographic variables was conducted for patients prescribed DOACs or warfarin. Incidence rates were calculated as the number of events per 100 person-years. Propensity score matching was used to control for potential confounders between DOACs and warfarin (apixaban vs warfarin, dabigatran vs warfarin, rivaroxaban vs warfarin) and DOACs versus DOACs (apixaban vs dabigatran, apixaban vs rivaroxaban, dabigatran vs rivaroxaban) [28]. Clinically-relevant covariates in the logistic regression model included demographics, Charlson comorbidity index score, comorbidities, baseline medications, and baseline hospitalization. Patients were matched using nearest neighbor matching with a caliper of 0.01 without replacement [29]. The balance of the covariates was checked based on standardized differences with a threshold of 10% [30]. Cox proportional hazard models were used to compare the risk of stroke/SE, MB, and MACE, among the matched cohorts.

A subgroup analysis was conducted per index dose (standard [apixaban 5mg, dabigatran 150mg, rivaroxaban 20mg]; lower dose [apixaban 2.5mg, dabigatran 75mg, rivaroxaban 10mg or 15mg]). The population was re-matched according to dose due to the significant demographic and clinical differences among patients prescribed low- and standard-dose DOACs. A sensitivity analysis was conducted for stroke/SE and MB where death was considered a competing risk using the Fine and Gray method [31]. A second sensitivity analysis was conducted where kidney failure was included in the PSM since renal function is a key variable in the choice of OAC for patients with NVAF and HF.

## Results

A total of 63,206 NVAF patients with HF meeting eligibility criteria were identified, of which 10,615 (16.8%) were prescribed apixaban, 4,297 (6.8%) were prescribed dabigatran, 15,921 (25.2%) were prescribed rivaroxaban, and 32,373 (51.2%) were prescribed warfarin (S1 Fig).

Before propensity score matching, apixaban and warfarin patients were of similar age, and dabigatran and rivaroxaban patients were younger. Dabigatran and rivaroxaban patients had lower CHA_2_DS_2_-VASc and HAS-BLED scores compared to apixaban patients. For apixaban, dabigatran, and rivaroxaban patients, 41.6%, 32.1%, and 45.0% (8.7% of rivaroxaban patients were on 10mg) were prescribed the lower dose, respectively (S2 Table). Table 1 shows the distribution of ICD-9-CM codes related to HF across different OAC cohorts. About 10% of patients had unspecified HF. More patients were classified as having diastolic HF (26–32%) than systolic HF (22–24%).

**Table 1:** Distribution of heart-failure-related ICD-9 codes by cohort.

	Apixaban		Dabigatran		Rivaroxaban		Warfarin	
	N	%	N	%	N	%	N	%
**Sample Size**	**10,615**		**4,297**		**15,921**		**32,373**	
**Heart Failure Codes**								
**428.0 (Congestive heart failure, unspecified)**	9,461	89.13%	3,838	89.32%	14,173	89.02%	29,513	91.17%
**428.1 (Left heart failure)**	682	6.42%	299	6.96%	992	6.23%	1,978	6.11%
**428.2 (Systolic heart failure)**	2,480	23.36%	936	21.78%	3,500	21.98%	7,647	23.62%
428.20 (Systolic heart failure, unspecified)	553	5.21%	197	4.58%	758	4.76%	1,779	5.50%
428.21 (Acute systolic heart failure)	579	5.45%	233	5.42%	892	5.60%	2,042	6.31%
428.22 (Chronic systolic heart failure)	1,355	12.76%	462	10.75%	1,719	10.80%	3,802	11.74%
428.23 (Acute on chronic systolic heart failure)	836	7.88%	297	6.91%	1,167	7.33%	2,686	8.30%
**428.3 (Diastolic heart failure)**	3,366	31.71%	1,153	26.83%	4,548	28.57%	9,211	28.45%
428.30 (Diastolic heart failure, unspecified)	1,128	10.63%	341	7.94%	1,521	9.55%	3,232	9.98%
428.31 (Acute diastolic heart failure)	820	7.72%	311	7.24%	1,154	7.25%	2,196	6.78%
428.32 (Chronic diastolic heart failure)	1,605	15.12%	512	11.92%	1,948	12.24%	4,096	12.65%
428.33 (Acute on chronic diastolic heart failure)	1,200	11.30%	335	7.80%	1,494	9.38%	3,234	9.99%
**428.4 (Combined systolic and diastolic heart failure)**	903	8.51%	288	6.70%	1,125	7.07%	2,695	8.32%
428.40 (Combined systolic and diastolic heart failure, unspecified)	154	1.45%	36	0.84%	190	1.19%	499	1.54%
428.41 (Acute combined systolic and diastolic heart failure)	139	1.31%	59	1.37%	205	1.29%	473	1.46%
428.42 (Chronic combined systolic and diastolic heart failure)	389	3.66%	125	2.91%	456	2.86%	1,038	3.21%
428.43 (Acute on chronic combined systolic and diastolic heart failure)	377	3.55%	112	2.61%	447	2.81%	1,184	3.66%
**428.9 (Heart failure, unspecified)**	1,109	10.45%	374	8.70%	1,524	9.57%	3,285	10.15%

Following propensity score matching, there were 10,570 apixaban-warfarin, 4,297 dabigatran-warfarin, 15,712 rivaroxaban-warfarin, 4,263 apixaban-dabigatran, 10,477 apixaban-rivaroxaban, and 4,297 dabigatran-rivaroxaban matched pairs. All baseline characteristics were balanced after propensity score matching. The mean follow-up was 6–8 months across all cohorts (Table 2, S3 Table).

**Table 2:** Characteristics in propensity score-matched DOAC vs warfarin users and DOAC vs DOAC.

	Apixaban	Warfarin	Dabigatran	Warfarin	Rivaroxaban	Warfarin	Apixaban	Dabigatran	Apixaban	Rivaroxaban	Dabigatran	Rivaroxaban
	Mean/%	Mean/%	Mean/%	Mean/%	Mean/%	Mean/%	Mean/%	Mean/%	Mean/%	Mean/%	Mean/%	Mean/%
**Sample Size**	**10,570**	**10,570**	**4,297**	**4,297**	**15,712**	**15,712**	**4,263**	**4,263**	**10,477**	**10,477**	**4,297**	**4,297**
Age*	80.44	80.42	78.7	78.95	79.34	79.36	78.65	78.76	80.36	80.31	78.7	78.83
**65–74**	25.92%	26.29%	33.58%	32.19%	30.64%	30.61%	34.15%	33.26%	26.20%	26.33%	33.58%	33.23%
**75–79**	19.53%	19.27%	21.32%	20.62%	21.44%	21.49%	20.99%	21.35%	19.77%	19.73%	21.32%	21.13%
**≥80**	54.55%	54.44%	45.10%	47.20%	47.93%	47.89%	44.85%	45.39%	54.03%	53.94%	45.10%	45.64%
Sex*												
**Male**	46.25%	46.20%	49.15%	48.43%	47.22%	46.99%	48.28%	49.05%	46.16%	46.06%	49.15%	48.41%
**Female**	53.75%	53.80%	50.85%	51.57%	52.78%	53.01%	51.72%	50.95%	53.84%	53.94%	50.85%	51.59%
Race*												
**White**	89.52%	89.61%	87.90%	88.13%	88.48%	88.42%	87.66%	87.99%	89.46%	89.32%	87.90%	87.46%
**Black**	5.72%	5.74%	6.47%	6.61%	6.14%	6.38%	6.43%	6.50%	5.73%	5.62%	6.47%	6.68%
**Hispanic**	1.44%	1.39%	1.72%	1.44%	2.01%	1.92%	1.69%	1.67%	1.45%	1.61%	1.72%	2.07%
**Other/Unknown**	3.32%	3.25%	3.91%	3.82%	3.37%	3.28%	4.22%	3.85%	3.36%	3.45%	3.91%	3.79%
US Geographic Region*												
**Northeast**	17.64%	17.73%	19.29%	19.22%	17.59%	17.72%	19.14%	19.26%	17.60%	17.66%	19.29%	17.45%
**Midwest**	21.94%	22.95%	22.39%	24.41%	22.96%	23.84%	23.01%	22.50%	21.93%	21.98%	22.39%	22.99%
**South**	45.23%	43.94%	39.96%	38.45%	42.11%	41.53%	39.43%	40.21%	45.21%	44.73%	39.96%	40.70%
**West**	15.11%	15.28%	18.20%	17.76%	17.18%	16.75%	18.25%	17.92%	15.18%	15.55%	18.20%	18.76%
**Unknown**	0.08%	0.10%	0.16%	0.16%	0.17%	0.17%	0.16%	0.12%	0.08%	0.09%	0.16%	0.09%
Proxy for Socioeconomic Status*												
**Low**	24.40%	24.50%	25.67%	24.58%	25.45%	25.32%	25.69%	25.64%	24.42%	24.49%	25.67%	25.60%
**Mid**	27.20%	27.09%	28.35%	28.97%	27.13%	27.60%	27.38%	28.24%	27.20%	27.07%	28.35%	28.37%
**High**	45.52%	45.61%	42.82%	43.22%	44.21%	43.78%	44.05%	43.02%	45.49%	45.55%	42.82%	42.68%
**Missing**	2.89%	2.80%	3.16%	3.23%	3.21%	3.30%	2.89%	3.10%	2.89%	2.89%	3.16%	3.35%
Medicaid dual-eligibility*	20.08%	20.51%	24.48%	23.43%	24.06%	23.82%	24.98%	24.09%	20.28%	20.68%	24.48%	24.16%
Part D Low-income subsidy*	31.74%	31.80%	37.26%	36.68%	34.70%	34.43%	37.77%	36.95%	31.88%	32.18%	37.26%	36.21%
**Baseline Comorbidity**												
Deyo-Charlson Comorbidity Index Score*	5.72	5.73	5.24	5.38	5.50	5.50	5.22	5.26	5.70	5.68	5.24	5.34
**CHA**_**2**_**DS**_**2**_**-VASc Score**	5.39	5.39	5.19	5.21	5.29	5.29	5.17	5.20	5.38	5.38	5.19	5.21
**HAS-BLED Score**^a^	3.74	3.72	3.50	3.51	3.60	3.60	3.49	3.50	3.73	3.71	3.50	3.54
Bleeding History*	28.53%	28.45%	25.79%	26.44%	28.46%	28.42%	25.03%	25.94%	28.50%	28.20%	25.79%	26.69%
Diabetes Mellitus*	47.86%	47.92%	48.08%	48.57%	48.22%	47.93%	47.64%	47.99%	47.75%	48.00%	48.08%	48.50%
Hypertension*	96.13%	95.69%	94.32%	94.41%	95.26%	95.36%	94.18%	94.49%	96.11%	96.07%	94.32%	94.72%
Liver Disease*	6.81%	6.83%	5.96%	5.70%	6.47%	6.35%	6.03%	5.98%	6.77%	6.84%	5.96%	5.91%
Renal Disease*	43.63%	43.83%	35.51%	37.84%	37.32%	37.83%	34.83%	35.77%	42.85%	42.25%	35.51%	36.44%
Myocardial Infarction*	15.77%	16.23%	14.24%	14.71%	16.07%	15.88%	13.86%	14.29%	15.75%	15.74%	14.24%	15.45%
Dyspepsia or Stomach Discomfort*	29.70%	29.07%	26.37%	26.46%	28.19%	28.07%	25.69%	26.53%	29.70%	29.45%	26.37%	26.67%
Peripheral vascular disease*	77.59%	77.76%	72.61%	73.56%	75.67%	76.08%	72.62%	72.88%	77.48%	77.42%	72.61%	74.40%
Prior Stroke/SE*	18.79%	18.82%	16.15%	16.41%	17.39%	17.43%	16.33%	16.26%	18.49%	18.59%	16.15%	16.27%
Transient Ischemic attack*	10.32%	10.34%	8.40%	8.87%	9.35%	9.34%	8.12%	8.47%	10.21%	10.18%	8.40%	8.40%
Anemia and Coagulation Defects*	48.52%	48.35%	43.82%	45.01%	45.39%	45.52%	42.86%	43.98%	48.14%	47.96%	43.82%	43.98%
Alcoholism*	0.25%	0.28%	0.40%	0.37%	0.33%	0.35%	0.38%	0.35%	0.25%	0.26%	0.40%	0.35%
**Peripheral Artery disease**	34.74%	36.12%	31.32%	32.51%	33.83%	35.27%	31.22%	31.41%	34.61%	34.49%	31.32%	33.16%
**Coronary Artery disease**	70.59%	70.00%	65.72%	66.21%	68.01%	68.45%	65.82%	65.99%	70.55%	69.61%	65.72%	67.09%
**Kidney Failure**	3.92%	7.48%	2.47%	6.49%	2.25%	6.59%	3.28%	2.49%	3.87%	2.41%	2.47%	1.91%
**Baseline Medication Use**												
ACEi/ARB*	65.66%	65.15%	67.91%	66.63%	67.24%	66.78%	67.82%	68.00%	66.00%	66.07%	67.91%	68.37%
Amiodarone*	19.79%	19.56%	18.01%	16.85%	17.62%	17.63%	18.18%	18.11%	19.78%	19.58%	18.01%	18.43%
Digoxin*	19.21%	19.33%	21.76%	20.64%	20.57%	20.30%	21.86%	21.67%	19.24%	19.37%	21.76%	21.15%
Diuretics*	77.35%	77.00%	77.36%	77.08%	75.77%	75.87%	77.06%	77.41%	77.29%	76.89%	77.36%	77.22%
Beta blockers*	59.56%	59.80%	57.11%	57.39%	59.08%	58.89%	57.26%	57.17%	59.62%	59.14%	57.11%	56.92%
Calcium Channel Blockers*	44.64%	44.12%	45.19%	44.45%	44.88%	44.66%	45.27%	45.09%	44.79%	44.37%	45.19%	44.71%
H2-receptor Blockers*	10.33%	10.20%	9.66%	9.31%	9.93%	10.01%	9.88%	9.64%	10.28%	10.19%	9.66%	9.70%
Proton Pump Inhibitors*	41.52%	40.77%	37.96%	38.03%	39.70%	39.48%	37.63%	38.17%	41.53%	41.37%	37.96%	39.19%
Statins*	65.71%	65.54%	60.46%	61.28%	62.96%	62.99%	60.36%	60.73%	65.48%	65.64%	60.46%	62.35%
Anti-platelets*	26.36%	25.83%	20.64%	19.69%	24.11%	23.69%	20.76%	20.78%	26.40%	26.03%	20.64%	22.92%
NSAIDs*	23.25%	23.13%	24.18%	22.39%	25.17%	24.71%	24.14%	24.02%	23.71%	23.79%	24.18%	25.13%
All-Cause Inpatient Admissions*	63.75%	64.16%	62.23%	62.93%	67.10%	67.25%	62.77%	62.21%	64.03%	64.13%	62.23%	64.11%
**Index Dose** ^b^^,^^c^^,^^d^												
**Lower Dose**	41.56%		32.14%		45.18%		35.02%	32.25%	41.17%	48.59%	32.14%	43.77%
**Standard Dose**	58.51%		67.93%		55.09%		65.07%	67.82%	58.90%	51.67%	67.93%	56.36%

ACEi: angiotensin-converting enzyme inhibitors; ARB: angiotensin-receptor blockers; CAD: coronary artery disease; CHA_2_DS_2_-VASc: congestive heart failure, hypertension, aged ≥75 years, diabetes mellitus, prior stroke or transient ischemic attack or thromboembolism, vascular disease, aged 65–74 years, sex category; HAS-BLED: hypertension, abnormal renal and liver function, stroke, bleeding, labile international normalized ratios, elderly, drugs and alcohol; NSAIDs: non-steroidal anti-inflammatory drugs; PAD: peripheral artery disease; SD: standard deviation; SE: systemic embolism

^a^ As the international normalized ratio value was not available in the data, a modified HAS-BLED score was calculated with a range of 0 to 8.

^b^ Lower dose: 2.5mg apixaban, 75mg dabigatran, 10mg or 15mg rivaroxaban. 1,375 (8.8%) of rivaroxaban-warfarin patients received 10 mg rivaroxaban; 950 (9.1%) and 349 (8.1%) received 10mg rivaroxaban in the apixaban-rivaroxaban and dabigatran-rivaroxaban cohorts

^c^ Standard dose: 5mg apixaban, 150mg dabigatran, 20mg rivaroxaban; After PSM, baseline characteristics were balanced between cohorts.

^d^ A few patients had both standard and lower doses on the index date.

*Covariates included in the propensity score matching

The Kaplan-Meier curves for cumulative incidence rates of stroke/SE, MB, and MACE are shown in S2, S3 and S4 Figs.

**Fig 1:**
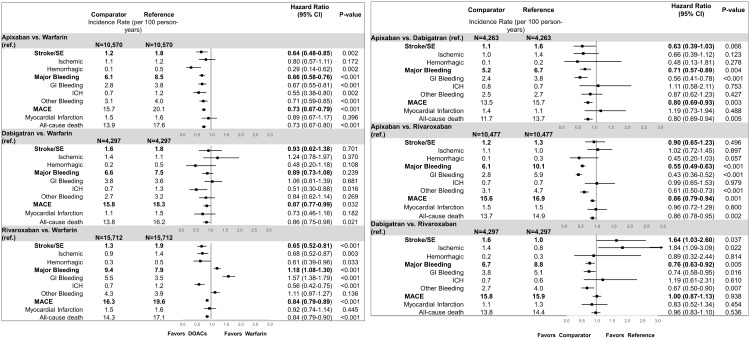
(a) Incidence rates and hazard ratios for patients with DOAC vs warfarin (b) incidence rates and hazard ratios for patients with DOAC vs DOACs. CI: confidence interval; GI: gastrointestinal; ICH: intracranial hemorrhage; MACE: major adverse cardiovascular event; SE: systemic embolism.

Apixaban was associated with a lower rate of stroke/SE (hazard ratio: 0.64, 95% confidence interval: 0.48–0.85), MB (hazard ratio: 0.66, 95% confidence interval: 0.58–0.76), and MACE (hazard ratio: 0.73, 95% confidence interval: 0.67–0.79) compared to warfarin. Compared to warfarin, dabigatran was associated with a lower rate of MACE (hazard ratio: 0.87, 95% confidence interval: 0.77–0.99), but no significant difference in stroke/SE (hazard ratio: 0.93, 95% confidence interval: 0.62–1.38) or MB (hazard ratio: 0.89, 95% confidence interval: 0.73–1.08). Compared to warfarin, rivaroxaban was associated with a lower rate of stroke/SE (hazard ratio: 0.65, 95% confidence interval: 0.52–0.81) and MACE (hazard ratio: 0.84, 95% confidence interval: 0.79–0.89); however, rivaroxaban was associated with an increased rate of MB (hazard ratio: 1.18, 95% confidence interval: 1.08–1.30; Fig 1).

Apixaban was associated with lower rate of MB (hazard ratio: 0.71, 95% confidence interval: 0.57–0.89) and MACE (hazard ratio: 0.80, 95% confidence interval: 0.69–0.93) compared to dabigatran. Apixaban patients had a lower rate of MB (hazard ratio: 0.55, 95% confidence interval: 0.49–0.63) and MACE (hazard ratio; 0.86, 95% confidence interval: 0.79–0.94) compared to rivaroxaban. Apixaban patients had a non-significantly lower rate of stroke/SE compared to dabigatran (hazard ratio: 0.63, 95% confidence interval: 0.39–1.03) and rivaroxaban patients (hazard ratio: 0.90, 95% confidence interval: 0.65–1.23). Compared to rivaroxaban, dabigatran was associated with a lower rate of MB (hazard ratio: 0.76, 95% confidence interval: 0.63–0.92) but a higher rate of stroke/SE (hazard ratio: 1.64, 95% confidence interval: 1.03–2.60; Fig 1). Dabigatran patients had a similar rate of MACE compared to rivaroxaban (hazard ratio: 1.00, 95% confidence interval: 0.87–1.13).

For the standard and lower dose subgroup analysis, the results were generally consistent with the main analysis, yet a few notable differences were observed within the apixaban-dabigatran and rivaroxaban-dabigatran comparisons (S4 Table). No significant differences were observed for stroke/SE, MB, and MACE between lower-dose apixaban and dabigatran users, while the main analysis found that apixaban was associated with a lower risk of MB and MACE, compared to dabigatran. Standard-dose dabigatran patients had significantly higher rates of MACE compared to rivaroxaban, as opposed to the main analysis findings where the rates were similar between the 2 cohorts.

The results for the competing risk analysis were shown to be consistent with the main analysis (S5 Table). When kidney failure was added to the PSM, the direction and the significance of effect is unchanged for most comparisons compared to the main analysis. As an exception, dabigatran had a similar risk of stroke/SE compared to rivaroxaban (HR: 1.19, 95% CI: 0.78–1.81), as opposed to a significantly increased risk as seen in the main analysis.

## Discussion

To the best of our knowledge, this real-world observational study is the largest Medicare study of NVAF patients diagnosed with HF, which evaluated the use of DOACs versus warfarin and DOACs versus DOACs. All DOACs were associated with a lower rate of MACE, driven by all-cause mortality. Differences were observed across DOACs versus warfarin in stroke/SE and MB events. Apixaban and rivaroxaban were both associated with a lower rate of stroke/SE, while apixaban use had a lower rate of MB, and rivaroxaban use had a higher rate of MB, compared to warfarin. Additionally, apixaban had lower rates of MB and MACE, compared to both dabigatran and rivaroxaban. The trends were relatively consistent when the patients were separated by standard- and lower-dose.

AF and HF prevalence has rapidly increased due to the aging US population; both conditions often occur concomitantly, as seen from randomized control trials and real-world studies [7–11,32]. These patients are at a high risk of ischemic stroke, myocardial infarction, and mortality [33]; therefore, examining a composite endpoint encompassing these severe outcomes is critical to profiling clinical burden in NVAF patients with HF. Additionally, in elderly patients, AF is associated with cognitive impairment, and HF has been reported to be a risk factor for Alzheimer’s disease [34–36]. With decreased cognitive ability, interference with self-care is prominent; hence, it becomes important to understand the effectiveness and safety of treatments in elderly patients with comorbid NVAF and HF. Patients with HF are an important subpopulation among elderly NVAF patients that warrant additional insights regarding the effectiveness and safety of DOACs in routine clinical practice.

Our study suggests that apixaban use was associated with a lower rate of stroke/SE and MB, compared to warfarin use, among NVAF patients diagnosed with HF, which is consistent with the ARISTOTLE main trial results. In the HF subgroup analysis of the ARISTOTLE trial, no significant interaction was found between the treatment effect of apixaban versus warfarin and the status of HF for stroke/SE (p = 0.21) or MB (p = 0.50) [8]. In the AVERROES trial, for the HF subgroup, compared to aspirin, apixaban had lower rates of stroke/SE while similar rates were observed for MB [37]. The current study found that rivaroxaban was associated with a lower risk of stroke/SE but a higher risk of MB. In the post-hoc ROCKET-AF analysis of patients with and without HF, the results were similar to those in the main population, where rivaroxaban was non-inferior to warfarin in the prevention of stroke/SE and MB; no significant interaction was observed between the treatment effect of rivaroxaban versus warfarin and HF status [5]. In the post-hoc RE-LY trial, there was no evidence of treatment heterogeneity according to HF status between dabigatran and warfarin users [7]. Both dabigatran 150mg and 110mg were non-inferior compared to warfarin for the reduction of stroke/SE risk. Our findings did not show significant reduction in stroke risk for dabigatran versus warfarin patients; however, the RE-LY trial examined 110 mg dabigatran as a lower dose, which is not available in the United States, whereas in our current sample, 32% of dabigatran patients were prescribed 75mg dabigatran.

Randomized control trials evaluating efficacy and safety of DOACs versus warfarin identified HF patients using varying lower ventricular ejection fraction thresholds [14]. The current analysis adds value by using a single HF criterion for all OAC users. Significant design differences exist between clinical trials and “real-world” settings; subsequently, many factors may lead to disparity in findings between these settings [38]. The stringent criteria and controlled environment under which patients are chosen, as opposed to claims-based studies which apply more relaxed selection criteria, yield a much larger sample size for analysis. Moreover, the ineffective management of warfarin patients who are better controlled in the trial environment may be a reason for the increase in outcome events in claims studies. In this analysis, outcomes were based on patient claims, and results were adjudicated in the clinical trials.

Few real-world observational studies in the United States have compared DOACs with warfarin among NVAF patients with HF or evaluated the treatment effects of NVAF patients with and without HF. Using Humedica electronic health records, Lin et al found that apixaban and dabigatran use in NVAF patients with HF was associated with a lower risk of MB compared to warfarin, and rivaroxaban use was associated with a higher risk of MB compared to warfarin [32]. Moreover, a retrospective cohort study among veterans showed that rivaroxaban patients had a similar risk of major and clinically-relevant non-major bleeding compared to warfarin patients [20]. In another study using the HealthCore claims database, in the overall NVAF population and in a subgroup of patients with HF, apixaban and dabigatran use were associated with a lower risk of MB; there was no significant difference between rivaroxaban and warfarin [9]. These studies have generally similar trends of bleeding reduction, as evidenced in our analyses.

There are no head-to-head randomized control trials comparing DOACs among NVAF patients; real-world comparative studies of DOACs exist and are increasing in number as the use of these medications becomes more widespread. These studies have shown that apixaban use is associated with similar or lower rates of stroke/SE and lower rates of MB compared to dabigatran and rivaroxaban [9,39–41]. In the HealthCore analysis that also evaluated various subgroups including patients with HF, apixaban and dabigatran users had a lower rate of MB compared to rivaroxaban in patients with and without HF. Also, apixaban patients had lower rates of MB compared to dabigatran in patients with HF [9]. These results are consistent with our findings. Although a large portion of patients in our study were prescribed the lower dose, the results of our analysis were generally consistent with prior RCTs and real-world observational studies.

A primary strength of this study is the large sample size, long follow-up period, and sufficient statistical power necessary to evaluate effectiveness and safety in the HF subpopulation. Our sample includes a nationally representative elderly population since nearly 2/3 Americans aged >65 years are enrolled in a fee-for-service Medicare health plan.

This study has some limitations that should be considered. First, this is a retrospective observational study; therefore, casual inference cannot be evaluated. Although the cohorts were matched using propensity scores, there may be residual confounding. Propensity score matching was conducted to match each of the OAC cohorts; thus, the results are not comparable across matched cohorts. With multiple comparisons, there is an increased risk for type I error and no further adjustments were made. Second, variables were based on International Classification of Diseases, 9^th^ Revision, Clinical Modification diagnosis and procedure, Healthcare Common Procedure Coding System, and National Drug Codes on billing claims; therefore, coding errors and lack of clinical accuracy may have introduced bias in the study. Next, laboratory values are not available in the Medicare claims data, so clinical parameters such as creatinine clearance level, international normalized ratio values, body mass index, and left ventricular ejection fraction information were not evaluated. Since there was a large portion of patients with unspecified HF, we are unable to draw meaningful conclusions about differences in HF type between cohorts. [42]. A FDA Mini-Sentinel evaluation, which aimed to describe the validity of algorithms used to detect HF using administrative and claims data sources, determined that current coding systems are unable to distinguish systolic/diastolic HF or detail a patient’s disease severity [43]. Despite the lack of reliability, the current distribution does not suggest any substantial difference in subtype of HF across OAC cohorts. The number of dabigatran users in the sample was much smaller compared to other OAC groups; thus, dabigatran comparisons led to larger confidence intervals and hence, those associations need to be interpreted with caution. Lastly, we were also unable to determine if patients were appropriately dose-adjusted according to dosing criteria. If doses are reduced inappropriately, worse clinical outcomes may occur [44].

## Conclusions

When compared to warfarin, apixaban use was associated with a lower rate of stroke/SE, MB, and MACE; dabigatran was associated with a lower rate of MACE; and rivaroxaban was associated with a lower rate of stroke/SE, and MACE and a higher rate of MB in this large Medicare population of NVAF patients diagnosed with HF. In addition, our DOACs versus DOACs analysis showed that apixaban was associated with lower rates of MB and MACE compared to dabigatran and rivaroxaban. Lastly, dabigatran use showed higher rates of stroke/SE, but lower rates of MB compared to rivaroxaban. Findings from this observational analysis provide important insights regarding the use of OAC therapy among patients with comorbid NVAF and HF in a real-world setting.

## Supporting information

S1 Fig Patient selection figure. No edoxaban patients were identified after applying the selection criteria. AF: atrial fibrillation; OAC: oral anticoagulant; VTE: venous thromboembolism.
(DOCX)

S2 Fig Cumulative incidence of stroke/SE in the propensity score-matched warfarin-NOAC and NOAC-NOAC cohorts. (DOCX)

S3 Fig Cumulative incidence of major bleeding in the propensity score-matched warfarin-NOAC and NOAC-NOAC cohorts. (DOCX)

S4 Fig Cumulative incidence of MACE in the propensity score matched warfarin-NOAC and NOAC-NOAC cohorts. (DOCX)

S1 Table ICD-9-CM Diagnosis and procedure codes for selection criteria and outcomes. AF: atrial fibrillation; VTE: venous thromboembolism.
(DOCX)

S2 Table Baseline characteristics of NVAF HF patients before PSM. ACEi: angiotensin-converting enzyme inhibitor; ARB: angiotensin-receptor blocker; CAD: coronary artery disease; CHA2DS2-VASc: congestive heart failure, hypertension, aged ≥75 years, diabetes mellitus, prior stroke or transient ischemic attack or thromboembolism, vascular disease, aged 65–74 years, sex category; HAS-BLED: hypertension, abnormal renal and liver function, stroke, bleeding, labile international normalized ratios, elderly, drugs and alcohol; NSAIDs: non-steroidal anti-inflammatory drugs; PAD: peripheral artery disease; PSM: propensity score matching; SD: standard deviation; SE: systemic embolism; SES: socioeconomic status. ^a^ As the INR value was not available in the data, a modified HAS-BLED score was calculated with a range of 0 to 8. ^b^ Reduced dose: 2.5mg apixaban, 75mg dabigatran, 10 or 15mg rivaroxaban [10mg rivaroxaban: 1,391 (8.7%)]. ^c^ Standard dose: 5mg apixaban, 150mg dabigatran, 20mg rivaroxaban.
(DOCX)

S3 Table Baseline characteristics in propensity score-matched DOAC vs warfarin and DOAC vs DOAC users. ACEi: angiotensin-converting enzyme inhibitors; ARB: angiotensin-receptor blockers; CAD: coronary artery disease; CHA2DS2-VASc: congestive heart failure, hypertension, aged ≥75 years, diabetes mellitus, prior stroke or transient ischemic attack or thromboembolism, vascular disease, aged 65–74 years, sex category; HAS-BLED: hypertension, abnormal renal and liver function, stroke, bleeding, labile international normalized ratios, elderly, drugs and alcohol; NSAIDs: non-steroidal anti-inflammatory drugs; PAD: peripheral artery disease; SD: standard deviation; SE: systemic embolism. ^a^ As the international normalized ratio value was not available in the databases, a modified HAS-BLED score was calculated with a range of 0 to 8. ^b^ Lower dose: 2.5mg apixaban, 75mg dabigatran, 10mg or 15mg rivaroxaban. (S3A Table): 1,375 patients (8.8%) received 10mg rivaroxaban in the rivaroxaban-warfarin cohort; (S3B Table): 950 (34.7%) and 349 (8.1%) received 10mg rivaroxaban in the apixaban-rivaroxaban and dabigatran-rivaroxaban cohorts. ^c^ Standard dose: 5mg apixaban, 150mg dabigatran, 20mg rivaroxaban; After PSM, baseline characteristics were balanced between cohorts. *Covariates included in the propensity score matching.
(XLSX)

S4 Table Incidence and hazard ratios of outcomes for patients with use of standard- and lower-dose DOACs vs warfarin and DOAC vs DOAC. ACM: all-cause mortality; CAD: coronary artery disease; CI: confidence interval; DOAC: direct oral anticoagulant; MACE: major adverse cardiac event; MB: major bleeding; MI: Myocardial infarction; PAD: peripheral artery disease; SE: systemic embolism; Lower dose: 2.5mg apixaban, 75mg dabigatran, 10 or 15mg rivaroxaban, Standard dose: 5mg apixaban, 150mg dabigatran, 20mg rivaroxaban. (S4 Table A) 1,381 patients received 10mg rivaroxaban in the rivaroxaban-warfarin cohort. (S4 Table B) 783 and 266 patients received 10mg rivaroxaban in the apixaban-rivaroxaban dabigatran-rivaroxaban matched cohorts, respectively.
(XLSX)

S5 Table Hazard ratios of stroke/SE and major bleeding among DOAC vs warfarin and DOAC vs DOAC, accounting for death as a competing event (sensitivity analysis). *The model incorporated death as a competing event. CI: confidence interval; DOACs: Direct acting oral anticoagulant; SE: systemic embolism.
(XLSX)

S6 Table Sensitivity analysis: Hazard ratios of stroke/SE and major bleeding among DOAC vs warfarin and DOAC vs DOAC, adjusting for kidney failure. CI: confidence interval; DOACs: Direct acting oral anticoagulant; SE: systemic embolism.
(DOCX)
