# Supplementary material for: Effectiveness and safety of oral anticoagulants in older adults with non-valvular atrial fibrillation and heart failure
Source: PLoS One. 2019 Mar 25;14(3):e0213614. doi: 10.1371/journal.pone.0213614 (PMC6433218; doi:10.1371/journal.pone.0213614)
Supplement: S1 Fig — No edoxaban patients were identified after applying the selection criteria. AF: atrial fibrillation; OAC: oral anticoagulant; VTE: venous thromboembolism. (DOCX) [file pone.0213614.s001.docx]

**S1 Fig. Patient selection figure.**

*
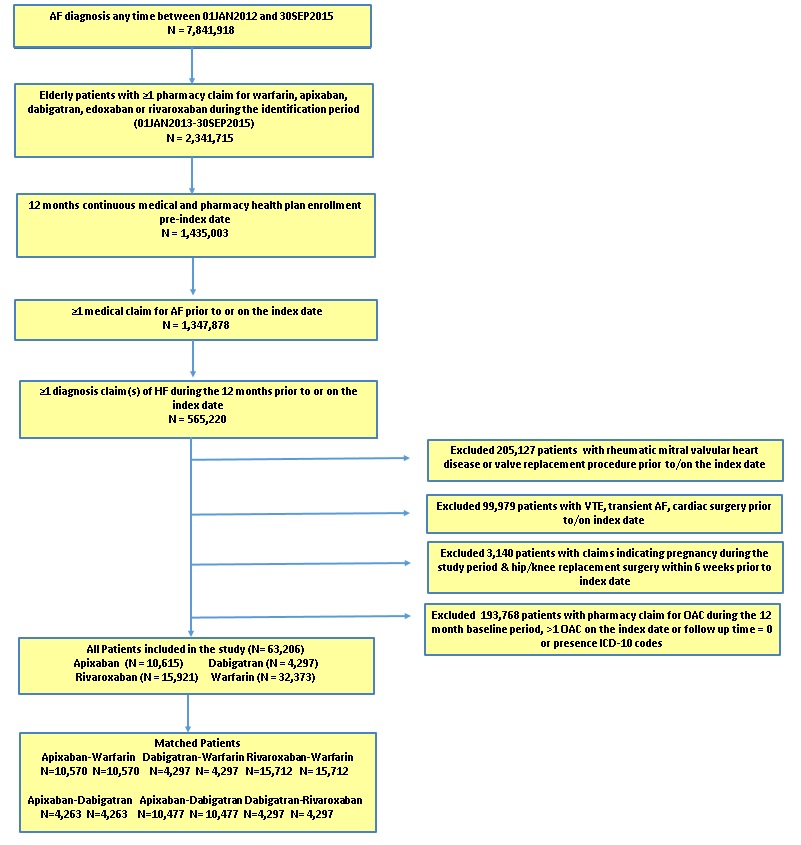
*

No edoxaban patients were identified after applying the selection criteria.

AF: atrial fibrillation; OAC: oral anticoagulant; VTE: venous thromboembolism
