# Supplementary material for: Effectiveness and safety of oral anticoagulants in older adults with non-valvular atrial fibrillation and heart failure
Source: PLoS One. 2019 Mar 25;14(3):e0213614. doi: 10.1371/journal.pone.0213614 (PMC6433218; doi:10.1371/journal.pone.0213614)
Supplement: S2 Fig — (DOCX) [file pone.0213614.s002.docx]

**S2 Fig. Cumulative incidence of stroke/SE in the propensity score-matched warfarin-NOAC and NOAC-NOAC cohorts.**

**
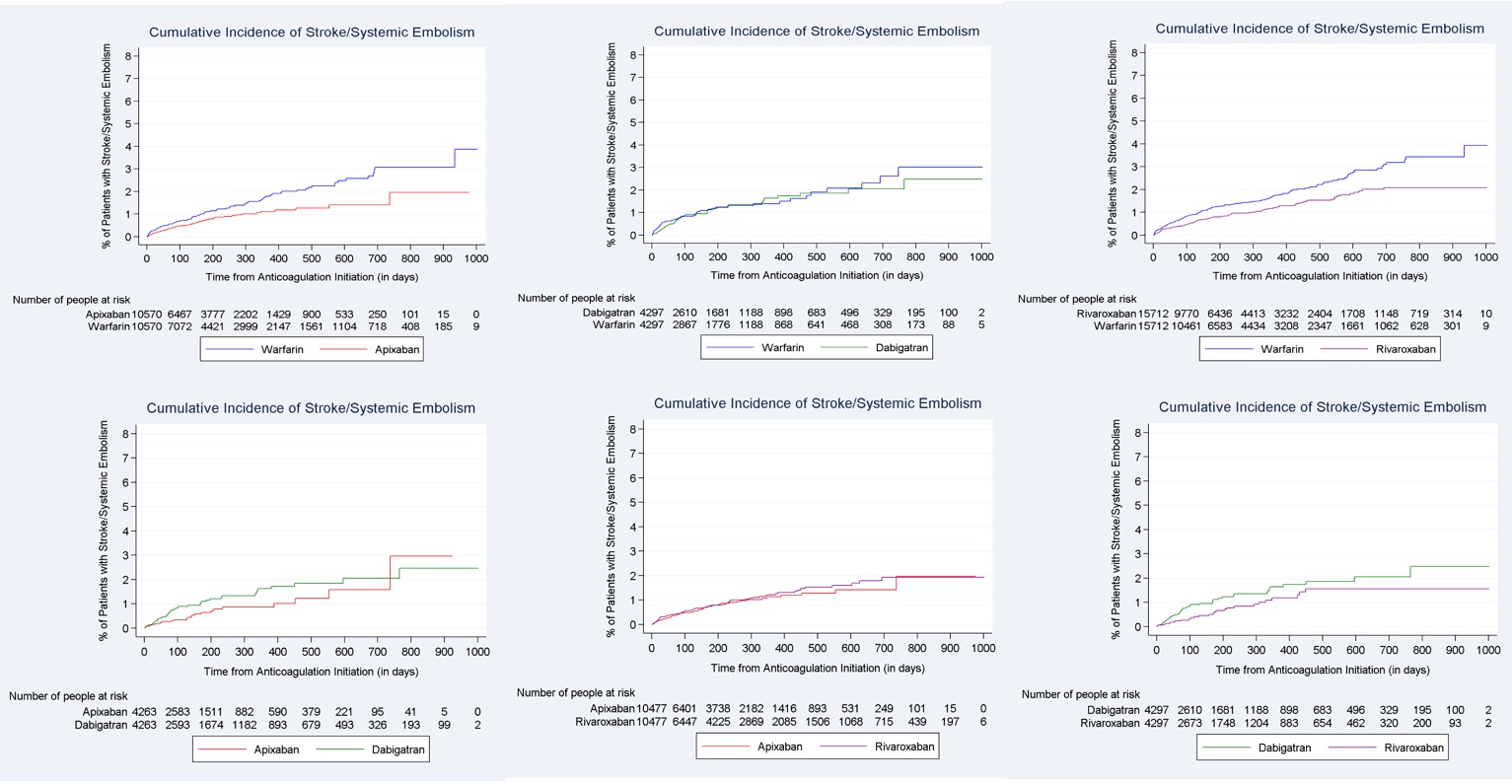
**
