# Supplementary material for: Effectiveness and safety of oral anticoagulants in older adults with non-valvular atrial fibrillation and heart failure
Source: PLoS One. 2019 Mar 25;14(3):e0213614. doi: 10.1371/journal.pone.0213614 (PMC6433218; doi:10.1371/journal.pone.0213614)

**S4 Fig. Cumulative incidence of MACE in the propensity score matched warfarin-NOAC and NOAC-NOAC cohorts.**


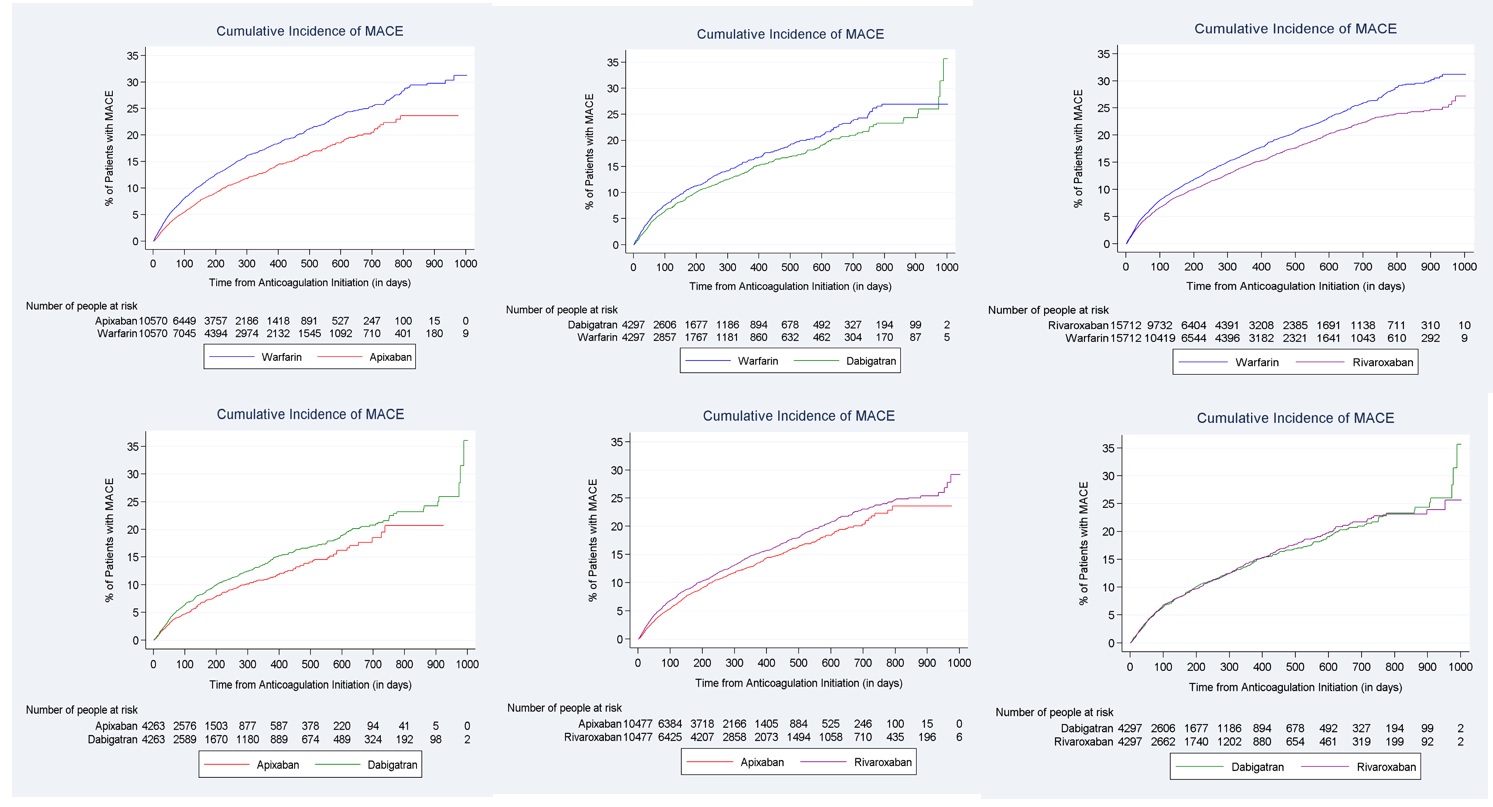

Supplement: S4 Fig — (DOCX) [file pone.0213614.s004.docx]
