# Supplementary material for: Effectiveness and safety of oral anticoagulants in older adults with non-valvular atrial fibrillation and heart failure
Source: PLoS One. 2019 Mar 25;14(3):e0213614. doi: 10.1371/journal.pone.0213614 (PMC6433218; doi:10.1371/journal.pone.0213614)
Supplement: S1 Table — AF: atrial fibrillation; VTE: venous thromboembolism. (DOCX) [file pone.0213614.s005.docx]

**S1 Table. ICD-9-CM Diagnosis and Procedure Codes for Selection Criteria and Outcomes.**

| **Diagnosis** | **ICD-9-CM Diagnosis and Procedure Codes** |
| --- | --- |
| **Selection Criteria** | |
| Atrial fibrillation | 427.31 |
| Valvular heart disease | 394.0, 394.1, 394.2, 394.9, 396.0, 396.1, 396.8, 396.9, 424.0, 745.xx |
| Heart valve replacement | V422, V433, 35.05-35.09, 35.20-35.28, 35.97 |
| VTE | 451-453, 671.3, 671.4, 671.9, 415.1, 673.2, 673.8 |
| Transient AF (Heart valve replacement/transplant, pericarditis, thyrotoxicity) | Pericarditis: 006.8, 017.9, 036.41, 074.21, 093.81, 098.83, 115.93, 390, 391, 392.0, 393, 411.0, 420.90, 420.91, 420.99, 423.0, 423.1, 423.2, 423.8, 423.9  Thyrotoxicity: 242.0, 242.1, 242.2, 242.3, 242.4, 242.8, 242.9 |
| Pregnancy | ICD-9-CM: 630-679, V22, V23, V24, V27, V28, V61.6, V61.7, 792.3, 796.5, 72-75.99  HCPCS: 59000-59350, 76801-76828, 83661-83664 |
| **Outcomes** | |
| Hemorrhagic Stroke | 430.xx-432.xx |
|  | Cases were excluded if traumatic brain injury (ICD-9-CM: 800-804, 850-854) was present during hospitalization. |
| Ischemic Stroke | 433.x1, 434.x1, 436 |
| Systemic Embolism | 444.x, 445.x |
| Major Gastrointestinal bleeding | 456.0, 456.20, 530.82, 531.0x, 531.2x, 531.4x, 531.6x, 532.0x, 532.2x, 532.4x, 532.6x, 533.0x, 533.2x, 533.4x, 533.6x, 534.0x, 534.2x, 534.4x, 534.6x, 535.01, 535.11, 535.21, 535.31, 535.41, 535.51, 535.61, 537.83, 562.02, 562.03, 562.12, 562.13, 568.81, 569.3, 569.85, 578.x |
|  | Procedure code: 44.43 |
| Major Intracranial Hemorrhage | 430, 431, 432.0, 432.1, 432.9, 852.0x, 852.2x, 852.4x, 853.0x, |
| Major Other hemorrhage | 285.1, 360.43, 362.43, 362.81, 363.61, 363.62, 363.72, 364.41, 372.72, 374.81, 376.32, 377.42, 379.23, 423.0x, 596.7x, 599.7x, 602.1x, 620.1, 621.4, 626.2, 626.5, 626.7, 626.8, 626.9, 719.1x, 782.7, 784.7, 784.8, 786.3x, 958.2, 997.02, 998.11 |
|  | Procedure code: 99.04 |
| Myocardial Infarction | 410.xx |

AF: atrial fibrillation; VTE: venous thromboembolism
