# Supplementary material for: Effectiveness and safety of oral anticoagulants in older adults with non-valvular atrial fibrillation and heart failure
Source: PLoS One. 2019 Mar 25;14(3):e0213614. doi: 10.1371/journal.pone.0213614 (PMC6433218; doi:10.1371/journal.pone.0213614)
Supplement: S2 Table — ACEi: angiotensin-converting enzyme inhibitor; ARB: angiotensin-receptor blocker; CAD: coronary artery disease; CHA2DS2-VASc: congestive heart failure, hypertension, aged ≥75 years, diabetes mellitus, prior stroke or transient ischemic attack or thromboembolism, vascular disease, aged 65–74 years, sex category; HAS-BLED: hypertension, abnormal renal and liver function, stroke, bleeding, labile international normalized ratios, elderly, drugs and alcohol; NSAIDs: non-steroidal anti-inflammatory drugs; PAD: peripheral artery disease; PSM: propensity score matching; SD: standard deviation; SE: systemic embolism; SES: socioeconomic status. a As the INR value was not available in the data, a modified HAS-BLED score was calculated with a range of 0 to 8. b Reduced dose: 2.5mg apixaban, 75mg dabigatran, 10 or 15mg rivaroxaban [10mg rivaroxaban: 1,391 (8.7%)]. c Standard dose: 5mg apixaban, 150mg dabigatran, 20mg rivaroxaban. (DOCX) [file pone.0213614.s006.docx]

**S2 Table. Baseline Characteristics of NVAF HF Patients Before PSM.**

|  | **Apixaban** | **Dabigatran** | **Rivaroxaban** | **Warfarin** |
| --- | --- | --- | --- | --- |
|  | **N/Mean** | **N/Mean** | **N/Mean** | **N/Mean** |
| **Sample Size** | **10,615** | **4,297** | **15,921** | **32,373** |
| **Age** | 80.5 (7.8) | 78.7 (7.6) | 79.3 (7.7) | 79.8 (7.8) |
| **65-74** | 2749 (25.9%) | 1443 (33.6%) | 4894 (30.7%) | 9348 (28.9%) |
| **75-79** | 2072 (19.5%) | 916 (21.3%) | 3436 (21.6%) | 9348 (19.7%) |
| **≥80** | 5794 (54.6%) | 1938 (45.1%) | 7591 (47.7%) | 9348 (51.5%) |
| **Sex** |  |  |  |  |
| **Male** | 4900 (46.2%) | 2112 (49.2%) | 7502 (47.1%) | 15783 (48.8%) |
| **Female** | 5715 (53.8%) | 2185 (50.9%) | 8419 (52.9%) | 16590 (51.3%) |
| **Race** |  |  |  |  |
| **White** | 9502 (89.5%) | 3777 (87.9%) | 14082 (88.5%) | 28403 (87.7%) |
| **Black** | 605 (5.7%) | 278 (6.5%) | 968 (6.1%) | 2485 (7.7%) |
| **Hispanic** | 152 (1.4%) | 74 (1.7%) | 336 (2.1%) | 512 (1.6%) |
| **Other/Unknown** | 356 (3.4%) | 168 (3.9%) | 535 (3.4%) | 973 (3.0%) |
| **US Geographic Region** |  |  |  |  |
| **Northeast** | 1866 (17.6%) | 829 (19.3%) | 2764 (17.4%) | 6703 (20.7%) |
| **Midwest** | 2319 (21.9%) | 962 (22.4%) | 3608 (22.7%) | 9868 (30.5%) |
| **South** | 4825 (45.5%) | 1717 (40.0%) | 6799 (42.7%) | 10713 (33.1%) |
| **West** | 1597 (15.0%) | 782 (18.2%) | 2720 (17.1%) | 5058 (15.6%) |
| **Unknown** | 8 (0.1%) | 7 (0.2%) | 30 (0.2%) | 31 (0.1%) |
| **Proxy for Socioeconomic Status** |  |  |  |  |
| **Low** | 2584 (24.3%) | 1103 (25.7%) | 4079 (25.6%) | 7332 (22.7%) |
| **Mid** | 2888 (27.2%) | 1218 (28.4%) | 4290 (27%) | 9731 (30.1%) |
| **High** | 4838 (45.6%) | 1840 (42.8%) | 7032 (44.2%) | 14362 (44.4%) |
| **Missing** | 305 (2.9%) | 136 (3.2%) | 520 (3.3%) | 948 (3.0%) |
| **Medicaid dual-eligibility** | 2126 (20.0%) | 1052 (24.5%) | 3858 (24.2%) | 7394 (22.8%) |
| **Part D Low-income subsidy** | 3370 (31.8%) | 1601 (37.3%) | 5548 (34.9%) | 10969 (33.9%) |
| **Baseline Comorbidity** |  |  |  |  |
| **Deyo-Charlson Comorbidity Index Score** | 5.7 (3.0) | 5.2 (2.9) | 5.5 (2.9) | 5.9 (3.1) |
| **CHA_2_DS_2_-VASc Score** | 5.4 (1.4) | 5.2 (1.4) | 5.3 (1.4) | 5.4 (1.4) |
| **HAS-BLED Score ^a^** | 3.7 (1.2) | 3.5 (1.2) | 3.6 (1.2) | 3.7 (1.3) |
| **Bleeding history (ICH, GI, other)** | 3021 (28.5%) | 1108 (25.8%) | 4501 (28.3%) | 10275 (31.7%) |
| **Hospitalized MI** | 843 (7.9%) | 309 (7.2%) | 1298 (8.2%) | 3303 (10.2%) |
| **Chronic Obstructive Pulmonary Disease** | 5234 (49.3%) | 2092 (48.7%) | 8054 (50.6%) | 16006 (49.4%) |
| **Cancer** | 1929 (18.2%) | 672 (15.6%) | 2769 (17.4%) | 5385 (16.6%) |
| **Diabetes Mellitus** | 5065 (47.7%) | 2066 (48.1%) | 7657 (48.1%) | 16666 (51.5%) |
| **Hypertension** | 10206 (96.2%) | 4053 (94.3%) | 15176 (95.3%) | 30526 (94.3%) |
| **Liver Disease** | 723 (6.8%) | 256 (6.0%) | 1036 (6.5%) | 2181 (6.7%) |
| **Renal Disease** | 4619 (43.5%) | 1526 (35.5%) | 5868 (36.9%) | 15560 (48.1%) |
| **Myocardial Infarction** | 1669 (15.7%) | 612 (14.2%) | 2547 (16.0%) | 5691 (17.6%) |
| **Dyspepsia or Stomach Discomfort** | 3157 (29.7%) | 1133 (23.4%) | 4503 (28.3%) | 8892 (27.5%) |
| **Peripheral vascular disease** | 8244 (77.7%) | 3120 (72.6%) | 12082 (75.9%) | 24296 (75.1%) |
| **Prior Stroke/SE** | 1992 (18.8%) | 694 (16.2%) | 2750 (17.3%) | 6616 (20.4%) |
| **Ischemic Stroke** | 1810 (17.1%) | 621 (14.5%) | 2486 (15.6%) | 5872 (18.1%) |
| **Hemorrhagic Stroke** | 174 (1.6%) | 46 (1.1%) | 208 (1.3%) | 568 (1.8%) |
| **Transient ischemic attack** | 1099 (10.4%) | 361 (8.4%) | 1496 (9.4%) | 3029 (9.4%) |
| **Anemia and Coagulation Defects** | 5141 (48.4%) | 1883 (43.8%) | 7183 (45.1%) | 16777 (51.8%) |
| **Alcoholism** | 26 (0.2%) | 17 (0.4%) | 53 (0.3%) | 86 (0.3%) |
| **Peripheral artery disease** | 3689 (34.8%) | 1346 (31.3%) | 5394 (33.9%) | 11657 (36.0%) |
| **Coronary artery disease** | 7504 (70.7%) | 2824 (65.7%) | 10862 (68.2%) | 21896 (67.6%) |
| **Baseline Medication Use** |  |  |  |  |
| **ACEs/ARBs** | 6978 (65.7%) | 2918 (67.9%) | 10750 (67.5%) | 20450 (63.2%) |
| **Amiodarone** | 2115 (19.9%) | 774 (18.0%) | 2850 (17.9%) | 5174 (16.0%0 |
| **Digoxin** | 2034 (19.2%) | 935 (31.8%) | 3274 (20.6%) | 6656 (20.6%) |
| **Diuretics** | 8216 (77.4%) | 3324 (77.4%) | 12062 (75.8%) | 24496 (75.7%) |
| **Beta blockers** | 6329 (59.6%) | 2454 (57.1%) | 9401 (59.1%) | 19025 (58.8%) |
| **Calcium Channel Blockers** | 4742 (44.7%) | 1942 (45.2%) | 7171 (45.0%0 | 14018 (43.3%) |
| **H2-receptor blockers** | 1097 (10.3%) | 415 (9.7%) | 1586 (10.0%) | 3204 (9.9%) |
| **Proton pump inhibitors** | 4414 (41.6%) | 1631 (38.0%) | 6362 (40.0%) | 12363 (38.19%) |
| **Statins** | 6982 (65.8%) | 2598 (60.5%) | 10033 (63.0%) | 20104 (62.1%) |
| **Anti-platelets** | 2823 (26.6%) | 887 (20.7%) | 3898 (24.5%) | 6774 (20.9%) |
| **NSAIDs** | 2490 (23.5%) | 1039 (24.2%) | 4109 (25.8%) | 6300 (19.5%) |
| **All-Cause Inpatient Admissions** | 6756 (63.7%) | 2674 (62.2%) | 10695 (62.7%) | 22090 (68.2%) |
| **Index Dose ^b,c^** |  |  |  |  |
| **Low Dose** | 4414 (41.6%) | 1381 (32.1%) | 7163 (45.0%) |  |
| **Standard Dose** | 6208 (58.5%) | 2919 (67.9%) | 8803 (55.3%) |  |
|  |  |  |  |  |

ACE: angiotensin-converting enzyme inhibitor; ARB: angiotensin-receptor blocker; CAD: coronary artery disease; CHA_2_DS_2_-VASc: congestive heart failure, hypertension, aged ≥75 years, diabetes mellitus, prior stroke or transient ischemic attack or thromboembolism, vascular disease, aged 65-74 years, sex category; HAS-BLED: hypertension, abnormal renal and liver function, stroke, bleeding, labile international normalized ratios, elderly, drugs and alcohol; NSAIDs: non-steroidal anti-inflammatory drugs; PAD: peripheral artery disease; PSM: propensity score matching; SD: standard deviation; SE: systemic embolism; SES: socioeconomic status

^a^ As the INR value was not available in the data, a modified HAS-BLED score was calculated with a range of 0 to 8.

^b^ Reduced dose: 2.5mg apixaban, 75mg dabigatran, 10 or 15mg rivaroxaban [10mg rivaroxaban: 1,391 (8.7%)].

^c^ Standard dose: 5mg apixaban, 150mg dabigatran, 20mg rivaroxaban
