# Supplementary material for: Effectiveness and safety of oral anticoagulants in older adults with non-valvular atrial fibrillation and heart failure
Source: PLoS One. 2019 Mar 25;14(3):e0213614. doi: 10.1371/journal.pone.0213614 (PMC6433218; doi:10.1371/journal.pone.0213614)
Supplement: S6 Table — CI: confidence interval; DOACs: Direct acting oral anticoagulant; SE: systemic embolism. (DOCX) [file pone.0213614.s010.docx]

**S6 Table. Distribution of heart failure-related ICD-9 codes by cohort in the study.**

|  | **Apixaban Cohort (Reference)** | | **Dabigatran Cohort** | | | **Rivaroxaban Cohort** | | | **Warfarin Cohort** | | |
| --- | --- | --- | --- | --- | --- | --- | --- | --- | --- | --- | --- |
|  | **N/Mean** | **%/SD** | **N/Mean** | **%/SD** | **Std**  **Difference** | **N/Mean** | **%/SD** | **Std**  **Difference** | **N/Mean** | **%/SD** | **Std**  **Difference** |
| **Sample Size** | **10,615** |  | **4,297** |  |  | **15,921** |  |  | **32,373** |  |  |
| **Heart Failure Codes** |  |  |  |  |  |  |  |  |  |  |  |
| **428.0 (Congestive heart failure, unspecified)** | 9,461 | 89.13% | 3,838 | 89.32% | 0.61 | 14,173 | 89.02% | 0.35 | 29,513 | 91.17% | 6.84 |
| **428.1 (Left heart failure)** | 682 | 6.42% | 299 | 6.96% | 2.13 | 992 | 6.23% | 0.80 | 1,978 | 6.11% | 1.30 |
| **428.2 (Systolic heart failure)** | 2,480 | 23.36% | 936 | 21.78% | 3.78 | 3,500 | 21.98% | 3.30 | 7,647 | 23.62% | 0.61 |
| 428.20 (Systolic heart failure, unspecified) | 553 | 5.21% | 197 | 4.58% | 2.90 | 758 | 4.76% | 2.06 | 1,779 | 5.50% | 1.27 |
| 428.21 (Acute systolic heart failure) | 579 | 5.45% | 233 | 5.42% | 0.14 | 892 | 5.60% | 0.65 | 2,042 | 6.31% | 3.63 |
| 428.22 (Chronic systolic heart failure) | 1,355 | 12.76% | 462 | 10.75% | 6.25 | 1,719 | 10.80% | 6.11 | 3,802 | 11.74% | 3.11 |
| 428.23 (Acute on chronic systolic heart failure) | 836 | 7.88% | 297 | 6.91% | 3.68 | 1,167 | 7.33% | 2.06 | 2,686 | 8.30% | 1.55 |
| **428.3 (Diastolic heart failure)** | 3,366 | 31.71% | 1,153 | 26.83% | 10.73 | 4,548 | 28.57% | 6.86 | 9,211 | 28.45% | 7.11 |
| 428.30 (Diastolic heart failure, unspecified) | 1,128 | 10.63% | 341 | 7.94% | 9.28 | 1,521 | 9.55% | 3.56 | 3,232 | 9.98% | 2.11 |
| 428.31 (Acute diastolic heart failure) | 820 | 7.72% | 311 | 7.24% | 1.85 | 1,154 | 7.25% | 1.81 | 2,196 | 6.78% | 3.63 |
| 428.32 (Chronic diastolic heart failure) | 1,605 | 15.12% | 512 | 11.92% | 9.38 | 1,948 | 12.24% | 8.40 | 4,096 | 12.65% | 7.14 |
| 428.33 (Acute on chronic diastolic heart failure) | 1,200 | 11.30% | 335 | 7.80% | 11.96 | 1,494 | 9.38% | 6.31 | 3,234 | 9.99% | 4.26 |
| **428.4 (Combined systolic and diastolic heart failure)** | 903 | 8.51% | 288 | 6.70% | 6.81 | 1,125 | 7.07% | 5.38 | 2,695 | 8.32% | 0.66 |
| 428.40 (Combined systolic and diastolic heart failure, unspecified) | 154 | 1.45% | 36 | 0.84% | 5.77 | 190 | 1.19% | 2.25 | 499 | 1.54% | 0.75 |
| 428.41 (Acute combined systolic and diastolic heart failure) | 139 | 1.31% | 59 | 1.37% | 0.55 | 205 | 1.29% | 0.19 | 473 | 1.46% | 1.30 |
| 428.42 (Chronic combined systolic and diastolic heart failure) | 389 | 3.66% | 125 | 2.91% | 4.24 | 456 | 2.86% | 4.51 | 1,038 | 3.21% | 2.52 |
| 428.43 (Acute on chronic combined systolic and diastolic heart failure) | 377 | 3.55% | 112 | 2.61% | 5.47 | 447 | 2.81% | 4.24 | 1,184 | 3.66% | 0.57 |
| **428.9 (Heart failure, unspecified)** | 1,109 | 10.45% | 374 | 8.70% | 5.93 | 1,524 | 9.57% | 2.92 | 3,285 | 10.15% | 0.99 |

Highlighted in green are standardized differences that are >10% among the cohorts. The Dabigatran, Rivaroxaban and Warfarin cohorts were compared with the Apixaban cohort.

The table above describes the distribution of ICD-9-CM codes related to heart failure (HF) across different OAC cohorts. It draws attention the high percentage of congestive HF patients classified as “unspecified”, which is consistent across cohorts. It is also unusual that the percentage of patients classified as diastolic HF (ICD-9-CM: 428.3) is higher than the percentage of patients classified as systolic HF (ICD-9-CM: 428.2), since the prevalence of systolic HF is commonly higher than diastolic HF in clinical practice.

That leads us to two initial conclusions regarding this distribution of ICD-9-CM codes. First, that we are not actually aware of the distribution of HF subtypes by cohort in this population because many patients were classified as unspecified HF, which precludes us from drawing meaningful conclusions, even though two of the standardized differences were slightly above 10% in the Dabigatran cohort. Second, that healthcare providers may be more judicious when using the ICD-9-CM coding system for classifying diastolic HF, since this is more uncommon than systolic.

An FDA Mini-Sentinel evaluation, which aimed to describe the validity of algorithms used to detect HF using administrative and claims data sources, reinforces the first conclusion above by stating that “current coding systems do not allow for algorithms that distinguish systolic and diastolic HF or that detail a patient’s disease severity” (Saczynski et al., 2012). Despite the lack of reliability, the current distribution does not suggest any substantial difference in subtype of HF across OAC cohorts.
